# Aging and wave-component latency delays in oVEMP and cVEMP: a systematic review with meta-analysis^☆^

**DOI:** 10.1016/j.bjorl.2016.12.006

**Published:** 2017-02-02

**Authors:** Ysa Karen dos Santos Macambira, Aline Tenório Lins Carnaúba, Luciana Castelo Branco Camurça Fernandes, Nassib Bezerra Bueno, Pedro de Lemos Menezes

**Affiliations:** a Universidade Estadual de Ciências da Saúde de Alagoas (UNCISAL), Audiologia, Maceió, AL, Brazil; b Universidade Federal de Alagoas (UFAL), Rede Nordeste de Biotecnologia (RENORBIO), Biotecnologia em Saúde, Maceió, AL, Brazil; c Universidade Estadual de Ciências da Saúde de Alagoas (UNCISAL), Maceió, AL, Brazil; d Universidade Federal de São Paulo (UNIFESP), Distúrbio da Comunicação, São Paulo, SP, Brazil; e Universidade Federal de Alagoas (UFAL), Maceió, AL, Brazil; f Universidade Federal de São Paulo (UNIFESP), Ciências, São Paulo, SP, Brazil; g Universidade de São Paulo (USP), Física aplicada à Medicina, São Paulo, SP, Brazil

**Keywords:** Cervical vestibular evoked myogenic potential, Ocular vestibular evoked myogenic potential, Elderly, Potencial evocado miogênico vestibular cervical, Potencial evocado miogênico vestibular ocular, Idosos

## Abstract

**Introduction:**

The natural aging process may result in morphological changes in the vestibular system and in the afferent neural pathway, including loss of hair cells, decreased numbers of vestibular nerve cells, and loss of neurons in the vestibular nucleus. Thus, with advancing age, there should be a decrease in amplitudes and an increase in latencies of the vestibular evoked myogenic potentials, especially the prolongation of p13 latency. Moreover, many investigations have found no significant differences in latencies with advancing age.

**Objective:**

To determine if there are significant differences in the latencies of cervical and ocular evoked myogenic potentials between elderly and adult patients.

**Methods:**

This is a systematic review with meta-analysis of observational studies, comparing the differences of these parameters between elderly and young adults, without language or date restrictions, in the following databases: Pubmed, ScienceDirect, SCOPUS, Web of Science, SciELO and LILACS, in addition to the gray literature databases: OpenGrey.eu and DissOnline, as well as Research Gate.

**Results:**

The n1 oVEMP latencies had a mean delay in the elderly of 2.32 ms with 95% CI of 0.55–4.10 ms. The overall effect test showed *p* = 0.01, disclosing that such difference was significant. The heterogeneity found was *I*^2^ = 96% (*p* < 0.001). Evaluation of p1 latency was not possible due to the low number of articles selected for this condition. cVEMP analysis was performed in 13 articles. For the p13 component, the mean latency delay in the elderly was 1.34 ms with 95% CI of 0.56–2.11 ms. The overall effect test showed a *p* < 0.001, with heterogeneity value *I*^2^ = 92% (*p* < 0.001). For the n23 component, the mean latency delay for the elderly was 2.82 ms with 95% CI of 0.33–5.30 ms. The overall effect test showed *p* = 0.03. The heterogeneity found was *I*^2^ = 99% (*p* < 0.001).

**Conclusion:**

The latency of oVEMP n1 wave component and latencies of cVEMP p13 and n23 wave components are longer in the elderly aged >60 years than in young adults.

## Resumo

**Introdução:**

O processo natural de envelhecimento pode resultar em mudanças morfológicas no sistema vestibular e na via neural aferente, incluindo perda de células ciliadas, diminuição do número de células do nervo vestibular e perda de neurônios no núcleo vestibular. Dessa forma, com o avanço da idade, deveria ocorrer diminuição nas amplitudes e aumento nas latências dos potenciais evocados miogênicos vestibulares (VEMP), principalmente o prolongamento da latência p13. Além disso, muitos artigos não encontraram diferenças significativas nas latências do VEMP, com o avanço da idade.

**Objetivo:**

Analisar se existem diferenças significativas para as latências do VEMP cervical (cVEMP) e do VEMP ocular (oVEMP) entre idosos e adultos.

**Método:**

Revisão sistemática com metanálise de estudos observacionais que comparam diferenças desses parâmetros entre idosos e adultos jovens, sem restrições de idiomas ou datas, nas seguintes bases de dados: Pubmed, *ScienceDirect*, Scopus, *Web of Science*, SciELO e Lilacs. Além das bases de literatura cinzenta: *OpenGrey.eu* e *DissOnline*, e ainda no *Research Gate*.

**Resultados:**

As latências n1 do oVEMP tiveram um atraso médio nos idosos de 2,32 ms com IC 95% 0,55–4,10 ms. O teste para o efeito geral obteve *p* = 0,01 e revelou que tal diferença foi significativa. A heterogeneidade encontrada foi I^2^ = 96% (*p* < 0,001). Avaliação da latência de p1 não foi possível devido ao baixo número de artigos selecionados para essa condição. A análise do cVEMP foi realizada com 13 artigos. Para o componente p13, o atraso médio para as latências dos idosos foi de 1,34 ms com IC 95% 0,56–2,11 ms. O teste para o efeito geral obteve *p* < 0,001; com valor da heterogeneidade I^2^ = 92% (*p* < 0,001). Para o componente n23, o atraso médio para as latências dos idosos foi de 2,82 ms com IC 95% 0,33–5,30 ms. O teste para o efeito geral obteve *p* = 0,03. A heterogeneidade encontrada foi I^2^ = 99% (*p* < 0,001).

**Conclusão:**

A latência do componente de onda n1 do oVEMP e as latências dos componentes de onda p13 e n23 do cVEMP são mais prolongadas em idosos com idade > 60 anos do que em adultos jovens.

## Introduction

The vestibular evoked myogenic potential (VEMP) is an objective, non-invasive examination with high-intensity auditory stimuli that assesses vestibular function integrity through the muscle reflex response.^1^, ^2^, ^3^

Recent advances in technology have allowed clinicians to assess the vestibular function capacity through the ocular (oVEMP) and cervical vestibular evoked myogenic potential (cVEMP).^1^, ^2^

OVEMP is a short-latency potential that evaluates the utriculo-ocular reflex (upper vestibular nerve),^3^ whereas cVEMP is a medium-latency potential^1^ that evaluates the saccular-colic reflex (lower vestibular nerve).^1^, ^2^, ^3^, ^4^, ^5^, ^6^ Thus, diseases that interfere with neural conduction from the inner ear, through the brainstem, the vestibulospinal tract and the second motor neuron, may interfere with the response. In view of this, the VEMP evaluates the final reflex; therefore, it cannot be used for the topographical diagnosis, but confirms or rules out the involvement of the affected pathway.^7^, ^8^, ^9^, ^10^

As a basic evaluation principle of any evoked potential, the time between the stimulus and the response is measured, classifying it as normal or altered based on the duration time and the morphology of the generated electric waves.^11^, ^12^, ^13^

The tracing obtained consists of two biphasic wave complexes. In the cVEMP, the first biphasic potential has a positive peak (P) with a mean latency of 13 milliseconds (ms), followed by a negative peak (N) with a mean latency of 23 ms, and it is called P13-N23; whereas the oVEMP shows a negative peak (N) with a mean latency of 10 ms, followed by a positive peak (P) with a mean latency of 15 ms, being called N10-P15.^4^, ^14^, ^15^, ^16^ The interaural difference of peak latency is associated with the neuronal conduction velocity, and the increase in this difference could be explained by the asymmetry in this velocity, common in neurological diseases.^17^, ^18^

Latency is the clinical parameter most often used in the analysis of VEMP responses, since it does not depend on stimulus intensity or the muscular tension level and has high reproducibility.^1^, ^19^

With the natural aging process, morphological changes may occur in the vestibular system and the afferent neural pathway, including loss of hair cells, decreased numbers of vestibular nerve cells and loss of neurons in the vestibular nucleus.^19^, ^20^, ^21^, ^22^ Therefore, with advancing age, there should be a decrease in amplitudes and an increase in latencies^22^ of these potentials, especially the prolongation of p13 latency. However, some authors report that VEMP latency cannot be affected by the otolytic function, but by the activation of the organ receptor.^22^ Additionally, many investigations did not find significant differences in VEMP latencies with advancing age.^21^, ^23^, ^24^, ^25^, ^26^, ^27^ Therefore, the aim of this study was to determine if there are significant differences regarding cVEMP and oVEMP latencies between the elderly and young adults.

## Methods

The devising of this systematic review sought to answer the following question: Do the elderly have different latency values of cervical and ocular vestibular evoked myogenic potentials than adults? Based on this question, the review is reported according to the items of the Preferred Reporting Items for Systematic Reviews and Meta-Analyses Statement (PRISMA). A protocol was published in the PROSPERO database^28^ (http://www.crd.york.ac.uk/PROSPERO), under registration number CRD42016046991.

### Search strategy

The strategy includes the descriptors (DECs and MESH) and Free terms (TL), based on the two first elements of PIC (Population, Interest, Context) present in the title, which consisted of: (*senile OR Age-related OR Aged OR Aging OR Ageing Effect OR Ageing OR older*) *AND* (*vestibular evoked myogenic potential OR vestibular potential OR VEMP OR Cervical evoked potential OR Ocular evoked potential OR* [*Vestibular AND evoked AND Potential*]). The complete strategy can be found in the supplementary material (Table 1).

**Table 1:** Literature search strategy, used for all databases.

*MEDLINE (via PubMed)*

*#1 E #2*
#1 (Cervical vestibular evoked myogenic potential) OR (myogenic potential) OR (vestibular potential) OR (Cervical evoked potential) OR (Ocular evoked potential) OR ((Vestibular) AND (Evoked potential)
#2 (senile) OR (related to aging) OR (elderly) OR (Aging) OR (Effect of aging) OR (Aging) OR (Elderly) OR (50 years old) OR (60 years old) OR (65 years old) OR (70 years old)

*ScienceDirect/ClinicalTrials.gov/LILACS/Scopus/Web of Science and other bases*
(Vestibular evoked myogenic potential OR vestibular potential OR VEMP OR Cervical evoked potential OR Ocular evoked potential OR (Vestibular Potential and evoked) AND (senile OR Related to aging OR Elderly ORL Aging OR Effect of aging OR Aging OR Elderly)

The searches were carried out between the months of July and August of 2016, and were revised in September of the same year. The following databases were searched: Pubmed, ScienceDirect, BVS (LILACS), SCOPUS, Circumpolar Health Bibliographic Database, SciELO and EMBASE, as well as the gray literature databases: OpenGrey.eu, DissOnline, The New York Academy of Medicine, as well as ReasearchGate. There was no manual search of the included articles and experts in the area were not contacted to avoid the risk of citation bias.^29^

### Eligibility criteria

The following were considered inclusion criteria: observational studies, with groups of elderly individuals, with age groups of 55 years or older, with control group, with latencies of ocular and/or cervical vestibular evoked myogenic potentials. Additionally, the potentials should be evoked by acoustic stimuli such as Click or 500 Hz Toneburst, with intensity between 90 and 105 dBNAn. Exclusion criteria were: conductive hearing loss, sensorineural hearing loss equal to or greater than 50 dB at any frequency, control group with age group containing subjects 55 years of age or older, vestibular, neural pathologies, diabetes, or Parkinson's disease. Articles that were repeated in different databases were also excluded. Finally, studies with at least the title and/or abstract in English were included, but there was no restriction regarding language or date of publication.

### Data extraction

During the selection process, the titles and abstracts of the obtained articles were independently evaluated by two researchers who were not blinded to the authors or journal title. Disagreements were resolved by discussion. In cases where there was no consensus, a third author was asked to make the final decision. The full texts of potentially eligible articles were acquired and analyzed in full.

The outcome sought in the studies was the mean latency values of the biphasic components for cVEMP and/or oVEMP and in the second assessment, associated with a dispersion measure.

Data were analyzed from published articles and authors were contacted for additional information. In addition to the outcome data, we also obtained the names of the authors, title, year of publication, country, age ranges of the groups, number of subjects in each group, monitored muscles and auditory examinations. A standard form for data storage was created based on the model used by Cochran.^30^

### Assessment of bias risk

The risk of bias was assessed according to the recommendations of the “Newcastle-Ottawa” manual and scale,^31^ adapted for cross-sectional observational studies. The quality of the study was independently evaluated by two researchers and the divergences were resolved by consensus. The maximum score to be reached was ten points and the evaluated scale items were: (1) representativeness of the sample; (2) sample size; (3) management of non-responses; (4) exposure calculation (risk factor); (5) comparability, to investigate whether individuals in different groups of outcomes are comparable, based on study design or analysis, control of confounding factors; (6) evaluation of results and (7) statistical test (Table 2).

**Table 2:** Newcastle-Ottawa Scale (adapted) for quality assessment of cross-sectional studies.

***Selection**: (Maximum of 5 stars)*
1. Sample representativeness:
a) Truly representative of the mean in the target population. * (All subjects or random sampling).
b) A little representative of the mean in the target population. * (Non-random sampling).
c) Group of selected users.
d) Description of the sampling strategy.
2. Sample size:
a) Justified and satisfactory.*
b) Not justified.
3. Non-responses:
a) Comparability between responses and non-responses is established, and the response rate is satisfactory.*
b) The response rate is not satisfactory, or the comparability between responses and non-responses is unsatisfactory.
c) Description of response rate or characteristics of responses and non-responses.
4. Exposure calculation (risk factor):
a) Validated measurement tool.**
b) Measurement tool not validated, but the tool is available or described.*
c) Description of the measurement tool.

***Comparability**: (Maximum of 2 stars)*
1. The objects in different result groups are comparable, based on the study design or analysis. Confounding factors are controlled.
a) The study considers the most important factor (select one).*
b) Study control for any additional factor.*

***Result**: (Maximum of 3 stars)*
1. Result assessment:
a) Independent blind evaluation.**
b) Record association.**
c) Study's own report.*
d) No description.
2. Statistical test:
a) The statistical test used to analyze the data are clearly described and adequate, and the association measurement is presented, including confidence intervals and the probability level (*p*-value).*
b) The statistical test is not appropriate, not described or incomplete

This scale was adapted from the Newcastle-Ottawa Quality Assessment Scale for cohort studies to perform a quality assessment of cross-sectional studies for the systematic review, “Are healthcare workers’ intentions to vaccinate related to their knowledge, beliefs, and attitudes? A systematic review.”

## Data analysis

The latency variation of the biphasic components for cVEMP and oVEMP of the two groups (elderly group and adult group) was compared by meta-analysis. For this purpose, a random effects model was used as a measure of the effect of the mean difference between the groups and as a statistical method of analysis. An *α* value of 0.05 was considered statistically significant. When it was not possible to obtain adequate data for analysis, Cochran's recommendations were followed.

The statistical heterogeneity between studies was tested using the Cochran's *Q* test and inconsistency was tested using the *I*^2^ test. A value of *p* < 0.10 was considered statistically significant. When necessary, study characteristics considered potential sources of heterogeneity were included in a subgroup analysis. Furthermore, in the case of heterogeneity, studies were removed, one by one, to investigate whether that particular study was the source of heterogeneity.

All analyses were performed using RevMan 5.3 software (Cochrane Collaboration).

## Results

### Included studies

**Figure 1:**
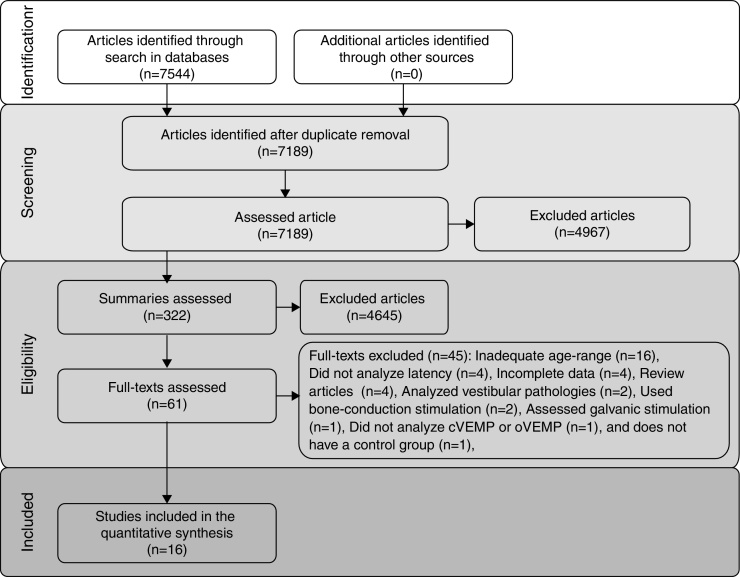
Flowchart of article search and selection.

The flow diagram that illustrates study search and selection is shown in Fig. 1. Of the 7544 titles considered relevant from the searches in these databases, 322 abstracts were read and, of those, 61 full texts were selected for reading in full. After reading, 41 articles were excluded, as they did not meet the eligibility criteria and four because they did not have sufficient data and their authors did not respond to the request for additional information (Table 3). Therefore, 16 full texts were included in the qualitative and quantitative analysis (Table 4). The latency means of young adults and elderly individuals of the meta-analyzed articles are shown in Table 5 (oVEMP) and Table 6 (cVEMP).

**Table 3:** Full texts excluded from the analysis.

Name	Location	Year	Reason	Name	Location	Year	Reason
Agrawal et al.	USA	2013	Age range	Maheu et al.	Canada	2015	Review article
Agrawal et al.	USA	2012	Age range	McCaslin et al.	USA	2016	Age range
Basta e Ernst	Germany	2007	Did not analyze latency	Meltem et al.	Turkey	2012	Age range
Beyazpınar et al.	Turkey	2016	Bone-conduction stimulation	Murofushi et al.	Japan	2010	Age range
Bigelow et al.	USA	2016	Vestibular pathology	Nguyen et al.	USA	2010	Age range
Bigelow et al.	USA	2015	Age range	Ochi and Ohashi	Japan	2003	Incomplete data^a^
Brantberg et al.	Norway	2007	Incomplete data^a^	Papathanasiou	Greece	2016	Review article
Chang et al.	Taiwan	2012	Galvanic stimulation	Papathanasiou	Greece	2013	Review article
Colebatch et al.	Australia	2013	Age range	Piker et al.	USA	2015	Did not analyze latency
Cosi et al.	Italy	1982	Did not analyze cVEMP/oVEMP	Piker et al.	USA	2013	Did not analyze latency
Dennis et al.	Australia	2014	Age range	Piker et al.	USA	2011	Age range
Derinsu et al.	Turkey	2009	Age range	Rosengren et al.	Australia	2011	Age range
Eleftheriadou et al.	Greece	2009	Age range	Sun et al.	USA	2014	Age range
Erbek et al.	Turkey	2014	Age range	Tourtillott et al.	Canada	2010	Age range
González-García et al.	Spain	2007	Incomplete data^a^	Tseng et al.	Taiwan	2010	Bone-conduction stimulation
Halmagyi and Curthoys	Australia	1999	Age range	Versino et al.	Italy	2015	Age range
Hong et al.	Korea	2008	Vestibular pathology	Walther et al.	Germany	2010	Age range
Isaradisaikul et al.	Thailand	2012	Age range	Walther LE et al.	Germany	2011	Age range
Iwasaki and Yamasoba	Japan	2015	Systematic review	Welgampola and Colebach	Australia	2001	Did not analyze latency
Kurtaran et al.	Turkey	2016	No control	Zahang et al.	China	2014	Age range
Layman et al.	USA	2015	Age range	Zapala and Brey	USA	2004	Age range
Li et al.	USA	2015	Incomplete data^a^	Zuniga et al.	USA	2012	Age range
Maes et al.	Belgium	2010	Age range				

a The authors were contacted but did not provide additional information until the submission of this article.

**Table 4:** Characteristics of included studies.

Authors	Year	Place	Groups of adults (years)	*N* (Elderly)	Intensity	Stimulus	Assessment
Akin et al.^23^	2011	USA	Group I (22–31), Group II (61–86)	24	90 dBNAn	TB 500 Hz	cVEMP
Asal^39^	2016	Egypt	Group II (25–35), Group V (>55)	10	95 dBNAn	TB 500 Hz	oVEMP
Basta et al.^20^	2005	Germany	Group I (20–40), Group III (60–76)	20	90 dBNAn	Tb 500 Hz	cVEMP
Guillén et al.^24^	2005	Spain	Group I (11–30), Group III (>60)	10	100 dBNAn	Click	cVEMP
Janky and Shepard^32^	2009	USA	Group II (20–29), Group V (>60)	10	98 dBNAn	TB 500 Hz/Click	cVEMP
Fei et al.^36^	2015	China	Group I (20–40), Group III (>60)	20	95 dBNAn	TB 500 Hz	Both^a^
Khan et al.^25^	2014	India	Group II (16–35), Group IV (>55)	9	100 dBNAn	TB 500 Hz	cVEMP
Kumar et al.^33^	2015	India	Young adults (21–40), Elderly (>60)	30	100 dBNAn	TB 500 Hz	oVEMP
Kumar et al.^34^	2010	India	Group I (21–30), Group V (>60)	30	99 dBNAn	Click	cVEMP
Lee et al.^37^	2008	Korea	Group II (20–29), [Group VI (60–69), Group VII (>70)]^b^	[21]	95 dBNAn	Click	cVEMP
Maleki et al.^35^	2014	Iran	Group I (19–26), Group II (>60)	31	95 dBNAn	TB 500 Hz	cVEMP
Mandal and Barman^26^	2009	India	Group I (20–30), [Group IV (60–70), Group V (70–80)]^2^	[21]	105 dBNAn	TB 500 Hz	cVEMP
Sarda et al.^40^	2016	India	Group I (20–30), Group V (60–70)	10	95 dBNAn	TP 500 Hz	cVEMP
Singh et al.^38^	2014	Germany	Group II (20–30), [Group VI (60–70), Group VII (>70)]^2^	[40]	105 dBNAn	TB 500 Hz	cVEMP
Su et al.^21^	2004	Taiwan	Group II (21–40), Group IV (>60)	20	95 dBNAn	Click	cVEMP
Tourtillott^27^	2009	USA	Young adults (20–30), Elderly [(65–74), (75–85)]^2^	[20]	95 dBNAn	TB 500 Hz	cVEMP

a cVEMP and oVEMP latencies were assessed.

b The groups were analyzed together, as the criterion chosen for the group was >55 years or >60 years.

**Table 5:** Mean and standard deviation of oVEMP n1 and p1 latencies, for young adults and for the elderly, per study.

Authors	Mean n1 latency (±SD) ms		Mean p1 latency (±SD) ms		Stimulus
	Young adult group	Elderly group	Young adult group	Elderly group	
Asal (2016)^39^	11.6 ± 0.7	11.8 ± 0.1	–	–	TB 500 Hz
Fei et al. (2015)^36^	16.0 ± 1.1	20.0 ± 3.1	25.5 ± 3.6	26.6 ± 3.9	TB 500 Hz
Kumar et al. (2015)^33^	12.0 ± 1.2	14.6 ± 2.1	16.1 ± 1.3	19.4 ± 2.2	TB 500 Hz

**Table 6:** Means and standard deviations of cVEMP p13 and n23 latencies, for young adults and for the elderly, per study.

Authors	Mean p13 latency (±SD) ms		Mean p23 latency (±SD) ms		Stimulus
	Young adult group	Elderly group	Young adult group	Elderly group	
Akin et al. (2011)^23^	15.6 ± 0.8	16.0 ± 1.6	23.2 ± 1.7	23.2 ± 2.0	TB 500 Hz
Fei et al. (2015)^36^	16.0 ± 1.1	20.0 ± 3.1	25.5 ± 3.3	26.6 ± 3.9	TB 500 Hz
Guillén et al. (2005)^24^	11.1 ± 0.1	12.1 ± 0.7	17.6 ± 1.2	20.7 ± 1.9	Click
Janky and Shepard (2009)^32^^a^	17.6 ± 3.3	15.2 ± 2.0	23.6 ± 2.3	22.6 ± 2.0	TB 500 Hz
Janky and Shepard (2009)^32^^a^	14.5 ± 2.5	17.4 ± 6.69^2^	20.7 ± 2.2	25.3 ± 10.12^b^	Click
Khan et al. (2010)^25^	11.0 ± 0.9	11.3 ± 1.7	17.3 ± 2.1	17.6 ± 2.2	TB 500 Hz
Kumar et al. (2010)^34^	11.4 ± 1.2	13.4 ± 1.5	19.2 ± 2.3	22.3 ± 2.0	Click
Lee et al. (2008)^37^	13.1 ± 1.6	16.2 ± 2.4	18.8 ± 1.8	21.7 ± 2.8	Click
Maleki et al. (2014)^35^	15.5 ± 1.2	16.4 ± 1.7	24.7 ± 1.8	24.0 ± 2.0	TB 500 Hz
Mandal and Barman (2009)^26^	14.3 ± 1.6	14.4 ± 2.3	21.0 ± 1.6	20.8 ± 2.9	TB 500 Hz
Sarda et al. (2016)^40^	16.5 ± 2.4	21.8 ± 2.9	25.1 ± 2.7	29.1 ± 5.0	TP 500 Hz
Singh et al. (2014)^38^	14.4 ± 0.7	17.8 ± 1.2	23.7 ± 0.6	27.3 ± 1.3	TB 500 Hz
Su et al. (2004)^21^	11.4 ± 0.8	11.9 ± 0.7	18.2 ± 1.3	19.2 ± 1.4	Click
Tourtillott (2009)^27^	16.2 ± 1.3	16.0 ± 1.4	24.6 ± 1.1	23.9 ± 2.6	TB 500 Hz

a It is the same study, which analyzed TB 500 Hz and clicks.

b Standard deviation was not provided and calculated.

Among the selected studies, only three assessed oVEMP. However, one of them did not have p1 latency data and, thus, the meta-analysis of this component was very compromised. On the other hand, 13 articles had mean and standard deviation data for the cVEMP latency components, p13 and n23, for the control group and for the elderly group. Of these, four studies found a significant difference between the groups, one of them found a significant difference for p13 and non-significant for n23, three studies were not clear whether there were differences and five affirmed that there were no differences between groups.

A total of 120 subjects were studied for the assessment of n1 and p1 latencies of oVEMP, 60 of which were elderly and 60 were young adults, and 326 subjects, of which 296 elderly and 326 young adults, were evaluated for cVEMP p13 and n23 latencies.

In Table 6, all data on means and standard deviations were provided except the standard deviations of p13 and n23 latencies of the elderly of one of the studies, when evoked by Clicks. In this case, the standard deviations were calculated by applying an international convention in which the quotient: mean/2.5 is used to find the standard deviation.

### Bias risk assessment

The analysis of the quality of the included articles and, consequently, of the risk of bias, is shown in Table 7.^21^, ^23^, ^24^, ^25^, ^26^, ^27^, ^32^, ^33^, ^34^, ^35^, ^36^, ^37^, ^38^, ^39^, ^40^ All included studies are characterized as observational and cross-sectional studies. In addition, in the final evaluation, all had a percentage of quality equal to or superior to 50% (5/10), whereas two of them obtained a maximum score of 70% (7/10).

**Table 7:** Quality of included articles, according to the “Newcastle–Ottawa” quality assessment scale.

Authors	Sample representativeness	Justified sample size^a^	Non-response rate	Exposure calculation	Comparability	Result assessment	Appropriate statistical test	Final assessment^b^
Akin et al. (2011)^23^	Not representative	No	8.4%	Validated tool	Yes	Their own report	Yes	6/10
Asal (2016)^39^	Not representative	No	40% (non-satisfactory)	Validated tool	Yes	Their own report	Yes	5/10
Basta et al. (2005)^20^	Not representative	No	0%	Validated tool	Yes	Their own report	Yes	6/10
Guillén et al. (2005)^24^	Not representative	No	0%	Validated tool	Yes	Their own report	Yes	6/10
Janky and Shepard (2009)^32^	Not representative	No	46.7% (non-satisfactory)	Validated tool	Yes	Their own report	Yes	5/10
Fei et al. (2015)^36^	Not representative	No	cVEMP 10%, oVEMP 5%	Validated tool	Yes	Their own report	Yes	6/10
Khan et al. (2014)^25^	Little representative	No	Unclear (per group)	Validated tool	Yes	Their own report	Yes	6/10
Kumar et al. (2015)^33^	Not representative	Yes	40% (non-satisfactory)	Validated tool	Yes	Two independent assessments	Yes	7/10
Kumar et al. (2010)^34^	Not representative	Yes	43% (non-satisfactory)	Validated tool	Yes	Two independent assessments	Yes	7/10
Lee et al. (2008)^37^	Not representative	No	0%	Validated tool	Yes	Their own report	Yes	6/10
Maleki et al. (2014)^35^	Not representative	Yes	Unclear (per group)	Validated tool	Yes	Their own report	Yes	5/10
Mandal e Barman (2009)^26^	Not representative	No	7.2%	Validated tool	Yes	Their own report	Yes	6/10
Sarda et al. (2016)^40^	Not representative	No	40% (non-satisfactory)	Validated tool	Yes	Their own report	Yes	5/10
Singh et al. (2014)^38^	Not representative	Yes	40% (non-satisfactory)	Validated tool	Yes	Their own report	Yes	6/10
Su et al. (2004)^21^	Not representative	No	40% (non-satisfactory)	Validated tool	Yes	Their own report	Yes	5/10
Tourtillott (2009)^27^	Not representative	No	0%	Validated tool	Yes	Their own report	Yes	6/10

Results shown as: points obtained/maximum score.

a Minimum criterion of *n* ≥ 30 (central limit theorem).

b Maximum 10-star score.

Only one study assessed the sample representativeness,^25^ as it was a normative study and analyzed all available subjects in a certain period. All other studies made choices per convenience group.

The satisfactory sample size of the elderly group was a concern of four studies,^33^, ^34^, ^35^, ^36^ which conform to the central limit theorem, with samples larger than 30 subjects. However, none of them performed calculations to estimate the size of their samples.

The non-response rate was satisfactory in 50% of all studies using validated tools for data collection and the comparability between the control group and the elderly group was also possible for all of them. The evaluation of the results was carried out in all the studies through their own reports, except in the two studies,^33^, ^34^ in which wave analysis was carried out by two independent professionals. Finally, all studies used appropriate statistical tests.

### Data analysis

As the studies are not randomized, the groups showed great discrepancy as early as in the first evaluation. Thus, to avoid the phenomenon of regression to the mean, it was necessary to analyze the variations between the final and initial latency values, as well as the standard deviation associated to these variations.

### oVEMP: n1 and p1 latencies

**Figure 2:**
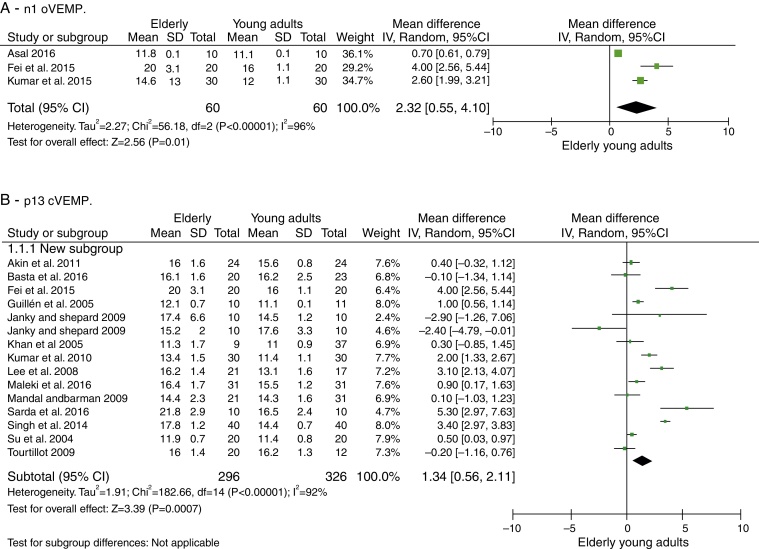
Meta-analysis: comparison of n1 oVEMP and p13 cVEMP latencies. (A) n1 oVEMP. (B) p13 cVEMP. * The study by Janky and Shepard 2009 appears twice, as it was carried out two different tests, one with click and another with TB.

The number of articles to be meta-analyzed for oVEMP n1 latencies was small, as there were only three of them.^33^, ^37^, ^38^ The mean delay of this component for the latencies of the elderly was 2.32 ms with 95% CI of 0.55–4.10 ms. The overall effect test showed *p* = 0.01; disclosing that such difference was significant. However, the heterogeneity *I*^2^ = 96%, with a *p* value <0.001 (Fig. 2A). Finally, due to the small number of selected studies, it was not possible to analyze the subgroups to understand the origins of this heterogeneity.

On the other hand, only two articles were found for the p1 component,^37^, ^38^ which considerably affected the analyses, as previously described, and made its study impossible.

### cVEMP: p13 and n23 latencies

The number of articles to be meta-analyzed for cVEMP components p13 and n23 latencies was quite encouraging. Thus, 13 were selected (described in Table 7).

For the p13 component, the mean delay for the latencies in the elderly was 1.34 ms with 95% CI of 0.56–2.11 ms. The overall effect test showed *p* < 0.001; disclosing that such a difference was significant. However, a heterogeneity value of *I*^2^ = 92% was found, with *p* < 0.001 (Fig. 2B).

**Figure 3:**
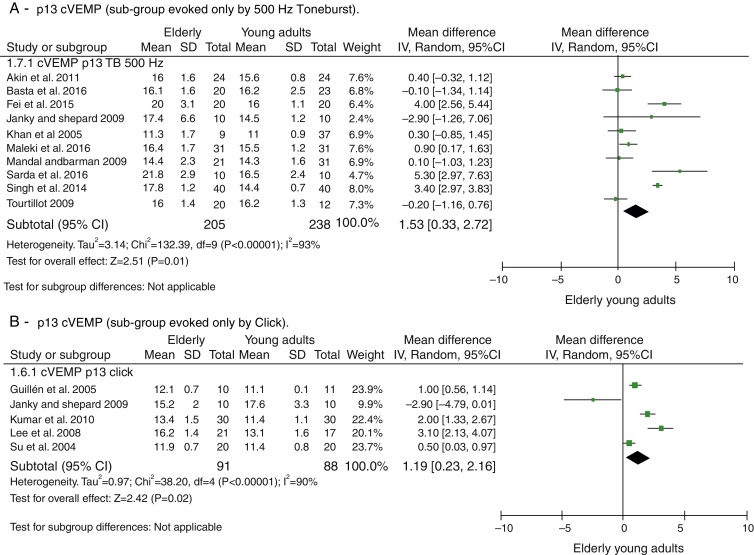
Meta-analysis: comparison of n13 cVEMP latencies, sub-groups evoked by 500 Hz Toneburst and only by Click. (A) p13 cVEMP (sub-group evoked only by 500 Hz Toneburst). (B) p13 cVEMP (sub-group evoked only by Click).

The attempts to analyze the subgroups were not successful in explaining heterogeneity. When dividing the groups by used stimuli to evoke cVEMP, Toneburst or Click, in both cases, it remained high and with *p* < 0.001, as can be seen in Fig. 3. The same was done for the stimulus intensity (up to 95 dBNAn and >95 dBNAn) and for the age ranges of the control groups (20–30 years and different <20–30 years), yet both evaluations were unsuccessful.

**Figure 4:**
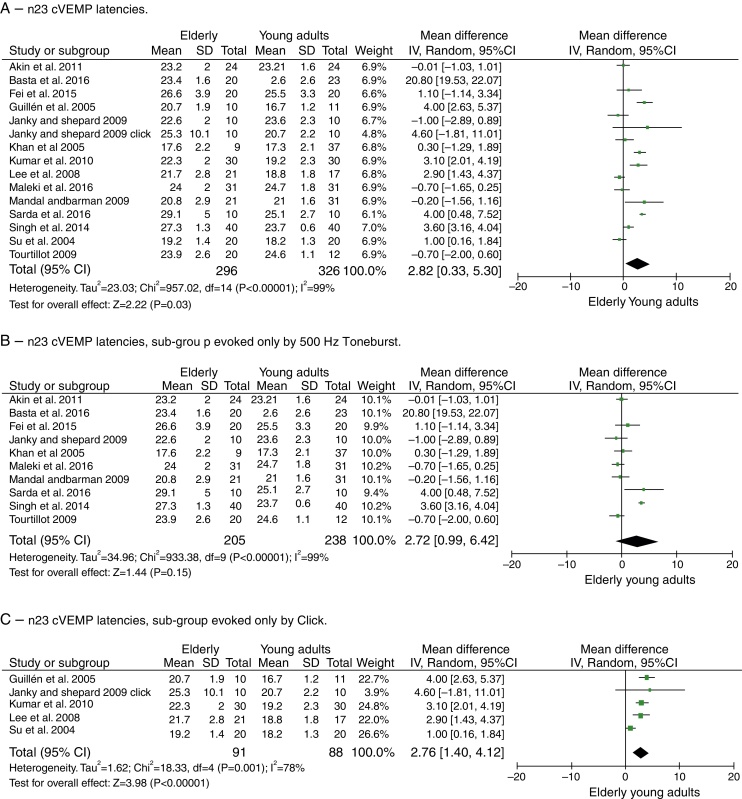
Meta-analysis: comparison of n23cVEMP latencies, n23 cVEMP sub-group evoked only by 500 Hz Toneburst and n23 cVEMP sub-group evoked only by Click. (A) n23 cVEMP latencies. (B) n23 cVEMP latencies, sub-group evoked only by 500 Hz Toneburst. (C) n23 cVEMP latencies, sub-group evoked only by Click.

For component n23, the mean delay for the latencies in the elderly was 2.82 ms with 95% CI of 0.33–5.30 ms. The test for the overall effect showed a *p* = 0.03; disclosing that the difference was significant. However, a high heterogeneity value of *I*^2^ = 99% was found, with *p* < 0.001 (Fig. 4A).

## Discussion

Due to the recent increase in the number of studies in the area of vestibular evoked myogenic potentials, this review highlight studies published between the years 2004 and 2016. The VEMPs were studied since the 1960s, but several centers only started to use it to evaluate the sacculo-colic reflex in the 1990s.^21^ Studies published at that time mostly reported on the methods used and studies in guinea pigs. From the year 2000 articles started to be published about the clinical applications, studies that involved pathologies aiming to assess the effectiveness of vestibular evoked myogenic potentials.^21^

Regarding the test protocols, the articles studied used strong intensity stimuli, ranging from 90 to 105 dBNAn; however, only two studies used the lowest intensity.^20^, ^23^ Most chose to evoke VEMP with Toneburst stimuli, corroborating the literature that recommends the use of Toneburst, because the threshold of saccular excitability is smaller when compared to the click, being more comfortable for the assessed subject, in addition to having a better definition of waves and greater response amplitude.^23^, ^24^, ^25^, ^26^, ^27^ Regarding the test frequency, the one most often used was 500 Hz, as it is the most often used clinically and has a more homogeneous and constant response.

The methodological quality of the studies was satisfactory, attaining at least 50% of the maximum score. The fact that only one study^25^ did not use convenience sampling is a fact of concern and very common in scientific studies, as they do not allow the creation of representative samples. On the other hand, all studies used validated tools for data collection and appropriate statistical tests,^20^, ^21^, ^23^, ^24^, ^25^, ^26^, ^27^, ^32^, ^33^, ^34^, ^35^, ^36^, ^37^, ^38^, ^39^, ^40^ which shows a greater concern with the quality of their quantitative analyses. A simple methodological adjustment can be observed in the studies, such as those performed in two articles^32^, ^33^ with wave analysis by two independent researchers, which helped them to increase the quality to the maximum found in the present systematic review.

According to the findings, the nV latency component of oVEMP and the p13 and n23 components of cVEMP were more delayed in the elderly than in young adults, as reported by all selected oVEMP studies^33^, ^37^, ^38^ and in five cVEMP studies,^34^, ^36^, ^38^, ^39^, ^40^ showing that it may be associated with the reduction in the number of neurons with advancing age, especially for subjects older than 60 years. In addition, advanced age and its association with the changes in the latency of the studied component due to aforementioned loss of neurons would have significant implications in the vestibular nucleus, which could be associated with balance deterioration in the elderly. Finally, it is quite reasonable to affirm, based on the results of the other components studied and if there were sufficient articles, that the p1 wave component of oVEMP will most likely also be delayed in the elderly.^33^, ^37^, ^38^

## Conclusion

The latency of oVEMP n1 wave component and the latencies of cVEMP p13 and n23 wave components are longer in the elderly aged 60 years or older than in young adults.

## Conflicts of interest

The authors declare no conflicts of interest.
